# 非小细胞肺癌中*EGFR*基因状态的检测对EGFR-TKIs疗效的预测价值

**DOI:** 10.3779/j.issn.1009-3419.2010.04.20

**Published:** 2010-04-20

**Authors:** 玉丽 王, 红雨 刘, 军 陈

**Affiliations:** 300052 天津，天津医科大学总医院，天津市肺癌研究所，天津市肺癌转移与肿瘤微环境重点实验室 Tianjin Key Laboratory of Lung Cancer Metastasis and Tumor Microenviroment, Tianjin Lung Cancer Institute, Tianjin Medical University General Hospital, Tianjin 300052, China

**Keywords:** EGFR-TKIs, Erlotinib, Gefitinib, PCR, FISH, IHC, EGFR-TKIs, Erlotinib, Gefitinib, PCR, FISH, IHC

## Abstract

近几年，表皮生长因子受体（epidermal growth factor receptor, EGFR）是肿瘤学药物研发的一个重要靶点。EGFR的小分子抑制剂（tyrosine kinase inhibitors, TKIs）成为目前非小细胞肺癌（non-small cell lung cancer, NSCLC）靶向治疗的热点。临床研究表明，EGFR-TKIs的临床疗效存在明显的个体差异，EGFR分子的存在状态影响TKI的疗效。EGFR外显子突变、EGFR拷贝数增加的患者对TKI的疗效较好，而存在KRAS突变患者提示对TKI耐药。

## Abstract (en)

Epidermal growth factor receptor (EGFR) is a major molecular for target therapy. The epidermal growth factor receptor tyrosine kinase inhibitors (TKIs), gefitinib (Iressa) and erlotinib (Tarceva), are two prospective agents towards non-small cell lung cancer (NSCLC). Multi-center clinical studies showed that there were obvious differences between indi- viduals by the treatment of EGFR-TKIs. EGFR status is the major factor that influences the outcome for the treatment by TKI. Exon mutations and amplification of EGFR molecule are the critical factors that predict good response to TKIs. On the other hand, KRAS mutations indicate the resistant to the TKIs.

肺癌是当今世界最常见的恶性肿瘤之一。其中非小细胞肺癌（non-small cell lung cancer, NSCLC）约占80%，而NSCLC中85%以上又都属中晚期肺癌而失去根治性手术治疗的机会。化疗在中晚期NSCLC治疗中占据主要地位，但疗效不尽如人意。近几年，以EGFR为靶点的分子靶向治疗在NSCLC的治疗中日渐突出。表皮生长因子受体酪氨酸激酶抑制剂（epidermal growth factor receptor tyrosine kinase inhibitors, EGFR-TKIs）主要包括吉非替尼（Gefitinib）和厄洛替尼（Erlotinib），是选择性酪氨酸激酶抑制剂。它们通过与EGFR结构域中高度保守的ATP结合位点竞争性结合EGFR，阻止酪氨酸残基磷酸化，从而阻止EGFR信号通路的传导，最终达到抑制肿瘤细胞的增生、侵袭、转移、血管生成并促进肿瘤细胞的凋亡。临床试验结果表明EGFR-TKIs生物利用度高、选择性强、耐受性好、不良反应轻微，具有显著的抗肿瘤活性。

EGFR-TKIs人群选择性强。多项临床试验^[1-3]^表明，亚裔、女性、无吸烟史、腺癌尤其是细支气管肺泡癌（bronchioloalveolar carcinoma, BAC）为Gefitinib和Erlotinib药物治疗的优势人群，通过这种优势人群的选择可使TKIs药物的有效性升高到40%以上。近年来，根据分子标志状态分层选择EGFR-TKIs受益人群是EGFR-TKIs优化治疗的重要环节。因此采用*EGFR*基因状态作为预测分子标志物，指导EGFR-TKIs的治疗，是一个重要而又有实际意义的问题。为初步认识NSCLC对靶向药物Gefitinib或Erlotinib敏感的分子机制，从而为预测其疗效提供有效的分子水平的预测指标，开创NSCLC治疗的新思路，本文就检测*EGFR*基因状态对EGFR-TKIs疗效预测的最新进展做一综述。

## 1 *EGFR*基因突变与TKIs疗效

*EGFR*基因是ErbB家族成员之一，位于染色体7p12-14区。*EGFR*基因酪氨酸激酶（EGFR TK）功能区由外显子18-24编码，在肺癌中*EGFR*基因突变90%以上集中在外显子18-21，其中特征性突变主要有：（1）外显子18点突变，719密码子甘氨酸错义突变为半胱氨酸（G719S）。（2）外显子19缺失，主要是编码氨基酸（KELREAT）序列缺失，这一缺失改变了EGFR受体ATP结合巢（ATP-binding cleft）的角度，增强了肿瘤细胞对小分子酪氨酸激酶抑制剂的敏感性^[3]^。（3）外显子20的点突变或碱基插入突变，点突变主要是第790位密码子出现C-T转换，引起EGFR蛋白中该位点的氨基酸由苏氨酸转变为甲硫氨酸（T790M），这一突变见于EGFR-TKIs治疗后复发者，突变使得肿瘤细胞对Gefitinib或Erlotinib产生抗性; 碱基插入突变出现在第770-775位密码子，存在8种不同的插入方式，插入的片段为3-9个碱基。这类插入突变的临床意义目前尚不清楚^[4, 5]^。（4）外显子21点突变，858位密码子亮氨酸置换成精氨酸（L858R），位于DGF序列附近，其作用使A-loop的稳定性增强，提高了肿瘤细胞对EGFR-TKIs的敏感性。

*EGFR*基因突变可显著提高EGFR-TKIs的临床反应率（表 1）。*EGFR*基因突变人群对Gefitinib或Erlotinib反应的阳性预测值（positive predictive value, PPV）在39.5%-95.8%范围内变化，EGFR野生型患者对Gefitinib或Erlotinib反应的阴性预测值（negative predictive value, NPV）在78.5%-100%范围内^[6-14]^。由于多项临床试验的条件不一致，PPV和NPV变化范围较大。Gupta等^[15]^对23项采用PCR检测*EGFR*突变的研究进行*meta*分析，评估了*EGFR*突变肺腺癌患者和EGFR-TKIs治疗反应的相关性。分析发现其中18项研究报道*EGFR*突变可增强患者对EGFR-TKI的反应，5项研究无发现相关性。

**1:** 采用PCR方法检测的EGFR突变对TKIs治疗的反应 Studies evaluating the predictive value of EGFR mutation and response to TKIs therapy

Author	Cases	EGFR(+)	EGFR-mutant patients responsive to TKI therapy	EGFR(-)	EGFR-nonmutant patients responsive to TKI therapy	PPV	NPV
Yoshida *et al*. (2009)^[7]^	100	48	46 (95.8%)	52	3 (5.8%)	95.8%	94.2%
Marcello *et al*. (2009)^[8]^	63	11	9 (81.8%)	52	5 (9.6%)	81.8%	90.4%
Miller *et al*. (2008) ^[9]^	81	18	15 (83.3%)	63	4 (6.3%)	83.3%	93.7%
Hirsch *et al*. (2007) ^[10]^	204	43	17 (39.5%)	113	8 (7.1%)	39.5%	92.9%
Sone *et al*. (2007) ^[11]^	59	17	10 (58.8%)	42	6 (14.2%)	58.8%	85.8%
Ichihara *et al*. (2007)^[12]^	98	38	25 (65.8%)	60	0	65.8%	100%
Sasaki *et al*. (2007) ^[13]^	54	26	19 (73.1%)	28	6 (21.4%)	73.1%	78.5%
Pugh *et al*. (2007) ^[14]^	39	8	7 (87.5%)	31	4 (12.9%)	87.5%	87.1%
Cappuzzo *et al*. (2007) ^[15]^	42	24	15 (62.5%)	13	3 (23.1%)	62.5%	76.9%
PPV: positive predictive value; NPV: negative predictive value.							

*EGFR*基因突变为接受靶向治疗NSCLC患者预后良好的分子标志。多数临床试验显示，EGFR-TKIs在难治性NSCLC患者的治疗中效果较好，患者的反应率达9%-19%，中位生存期（median survival time, MST）为6个月-8个月; 其中*EGFR*突变人群反应率可达到75%-90%，中位生存期提高至12个月-23个月^[16]^。IPASS（Iressa panAsian study）研究近期公布的数据显示：在突变人群中，Gefitinib治疗组无进展生存期（progression-free survival, PFS）长于CP方案（顺铂和环磷酰胺）治疗组（HR=0.48, *P* < 0.000 1），在突变阴性人群中则相反; 亚洲学者的IPASS研究^[17]^显示*EGFR*突变与PFS改善和肿瘤缓解显著相关。TRUST（Tarceva Lung Cancer Survival Treatment）研究最新数据^[18]^表明所有患者的疾病控制率（disease control rate, DCR）为69%，即使用厄洛替尼可使70%左右的患者临床获益，中位PFS为14.3周，1年生存率为38.6%，不良反应发生率低，患者耐受性良好; 亚组分析表明与EGFR野生型患者相比，*EGFR*基因突变患者的OS和PFS较长[风险比（hazard ratio, HR）分别为0.32和0.43，*P*值分别为0.013和0.016]，有效率较高（46% vs 7%，*P*=0.000 43）。大量的科学实验及临床研究表明：*EGFR*突变患者接受Gefitinib或Erlotinib治疗的有效率较高。*meta*分析证实*EGFR*突变为Gefitinib疗效的分子预测标志^[15]^。因此*EGFR*突变是预测EGFR-TKIs治疗有效率强有力的预测因素，肺癌病人接受靶向治疗之前应进行*EGFR*基因突变检测。

不同的EGFR突变类型预示EGFR-TKIs临床治疗效果的差异。Jackman等^[19]^对*EGFR*基因突变NSCLC患者进行的亚组分析结果表明，EGFR外显子19缺失患者总生存期及到进展/死亡时间都长于EGFR外显子21 L858R点突变者，且前者在症状改善方面也优于后者。

## 2 *EGFR*基因扩增

通过荧光原位杂交检测*EGFR* 基因拷贝数变化。*EGFR* 基因FISH（+）患者对TKIs的临床有效率高于FISH（-）（表 2）。PPV和NPV分别在30. 7%70%、53.3%-94.4%范围内变化^[7, 9-14, 20]^。Gupta等^[15]^通过*meta*分析16项研究，发现9项研究证实在肺腺癌患者中FISH检测的*EGFR*基因的拷贝数与EGFR-TKIs的疗效存在相关性，7项研究未得出这个结论，*meta*分析结果显示FISH（+）是TKIs有效的预测因子。

**2:** 采用FISH方法检测的EGFR扩增对TKIs治疗的反应 Studies evaluating the predictive value of EGFR amplification and polysomy and response to TKI therapy

Author	Cases	EGFR(+)	EGFR-mutant patients responsive to TKI therapy	EGFR(-)	EGFR-nonmutant patients responsive to TKI therapy	PPV	NPV
Varella-Garcia *et al*. (2009)^[21]^	44	29	19 (65.5%)	15	4 (26.7%)	65.5%	73.3%
Marcello *et al*. (2009) ^[8]^	54	12	6 (50%)	42	6(14.3%)	50%	85.7%
Hirsch *et al*. (2007) ^[10]^	204	59	20 (33.9%)	124	7 (5.6%)	33.9%	94.4%
Sone *et al*. (2007) ^[11]^	59	26	8 (30.7%)	28	6 (21.4%)	30.7%	78.6%
Sasaki *et al*. (2007)^[13]^	27	12	7 (58.3%)	15	7 (46.7%)	58.3%	53.3%
Pugh *et al*. (2007)^[14]^	39	10	7 (70%)	29	4 (13.8%)	70%	86.2%
Cappuzzo *et al*. (2007) ^[15]^	42	25	17 (68%)	11	1 (9.1%)	68%	90.9%

研究^[21]^证实*EGFR*基因拷贝数增加是EGFR-TKIs临床疗效的预测因子，为预后良好的分子标志。Schneider等^[22]^证实*EGFR*基因FISH（+）组对EGFR-TKIs反应率为17%，FISH（-）组仅为6%，且FISH（+）组PFS和OS明显增加; EGFR拷贝数增多与对Gefitinib的反应、DFS、OS相关^[23, 24]^;Hirsch等^[25]^发现*EGFR*基因扩增可增强Erlotinib的疗效：*EGFR*基因FISH（+）和FISH（-）组患者的生存期无差别，但FISH（+）组患者的进展/死亡时间明显延长。Varella-Garcia等^[26]^发现日本人群中EGFR FISH（+）率高，但对EGFR-TKIs疗效预测能力低于西方国家人群。总的来说，*EGFR*基因拷贝数可作为一个预后因素。*EGFR*基因拷贝数变化可以作为一个病理相关的EGFR临床意义的补充，不宜作为一个生存的预后因素^[27]^。

## 3 EGFR蛋白表达

EGFR蛋白过表达对TKIs临床有效率的预测观点不一（表 3）。Dziadziuszko等^[28]^证实EGFR蛋白过表达不能提高Gefitinib临床总体反应率，多项研究^[7, 8, 28]^报道EGFR蛋白过表达和EGFR-TKIs应答有相关性。

**3:** 采用IHC方法检测的EGFR过表达对TKIs治疗的反应 Studies evaluating the predictive value of EGFR overexpression and response to TKI therapy

Author	Cases	EGFR(+)	EGFR-mutant patients responsive to TKI therapy	EGFR(-)	EGFR-nonmutant patients responsive to TKI therapy	PPV	NPV
Marcello *et al*. (2009)^[8]^	59	45	11 (24.4%)	14	1 (7.1%)	24.4%	92.9%
Hirsch *et al*. (2007)^[10]^	204	121	27 (22.3%)	82	82 (4.9%)	22.3%	95.1%
Dziadziuszko *et al*. (2007) ^[29]^	82	58	12 (20.7%)	20	4 (20%)	20.7%	80%
Cappuzzo *et al*. (2007)^[15]^	42	23	13 (56.5%)	11	4 (36.4%)	56.5%	63.6%

Ohsaki等^[29]^研究290例NSCLC患者标本，124例为EGFR蛋白过表达，发现EGFR过表达和预后差相关（*P*=0.024 0）; EGFR蛋白过表达对患者预后有预测价值，然而EGFR蛋白表达和EGFR-TKIs反应无相关性^[30]^。Rusch等^[31]^未发现EGFR蛋白过表达和预后的相关性; 且EGFR蛋白过表达和EGFR-TKIs反应无相关性^[32]^。EGFR蛋白表达对预后的重要性仍存在争议^[33, 34]^。

## 4 *EGFR*基因突变、扩增、蛋白表达的相关性

研究^[35-37]^证实*EGFR*基因突变、扩增、蛋白过表达三者有显著的相关性。El-Zammar等^[38]^分别采用直接测序、FISH和免疫组织化学检测49例样本的*EGFR*基因变异，研究发现5例*EGFR*基因突变样本有4例FISH（+）和3例IHC（+），15例IHC（+）样本有13例FISH（+），因此应联合检测同一靶点基因不同状态的敏感性，从而选择更合适的治疗人群。

## 5 *KRAS*基因突变

*KRAS*基因是EGFR下游的关键调节分子，NSCLC中*KRAS*基因突变约占20%。Raponi等^[39]^报道NSCLC中*KRAS*基因突变对抗EGFR治疗的阴性预测值为97%，因此*KRAS*基因突变可用于排除NSCLC患者的抗-EGFR治疗，在EGFR-TKIs治疗前，*KRAS*基因突变作为常规检测指标; *KRAS*基因突变常提示患者预后不良，并对Gefitinib和Erlotinib的治疗有抗性，*KRAS*基因突变与肺癌患者对TKIs药物的原发耐药有关^[40-43]^。*KRAS*基因突变是EGFR靶向治疗的负性预测因子。

## 6 问题与展望

NSCLC的靶向治疗中，EGFR-TKIs应用最为广泛，且肿瘤取得缓解、生存期延长，生活质量和疾病相关症状改善，且耐受性好，显示出了较好的疗效与安全性。对女性、亚裔、非吸烟以及腺癌尤其是BAC患者等临床受益人群疗效更为明显。然而，如何根据分子基因指标对肺癌预后或疗效的预测进行分子分型，选择个体化的治疗方案，是提高NSCLC患者的治疗水平、延长患者生存的关键。EGFR突变、EGFR FISH/IHC等作为EGFR-TKIs临床应用的预测及预后候选分子标志成为目前的研究热点，*EGFR*基因状态对EGFR-TKIs疗效的预测仍需大样本量的临床实验进一步验证。随着靶向治疗研究的深入，相信会有更多的分子基因指标指导我们进行个体化治疗，如何选择有限数目基因作为分子标志用于临床试验或指导NSCLC患者选择用药为今后研究的重点。
